# Retraction: Down-regulation of the radiation-induced pEGFR^Thr654^ mediated activation of DNA-PK by Cetuximab in cervical cancer cells

**DOI:** 10.1039/d1ra90037d

**Published:** 2021-01-26

**Authors:** Laura Fisher

**Affiliations:** Royal Society of Chemistry Thomas Graham House, Science Park, Milton Road Cambridge CB4 0WF UK advances-rsc@rsc.org

## Abstract

Retraction of ‘Down-regulation of the radiation-induced pEGFR^Thr654^ mediated activation of DNA-PK by Cetuximab in cervical cancer cells’ by Yunxiang Qi *et al.*, *RSC Adv.*, 2020, **10**, 1132–1141, DOI: 10.1039/C9RA04962B.

The Royal Society of Chemistry hereby wholly retracts this *RSC Advances* article due to concerns with the reliability of the data. The images in the article were screened by an image integrity expert. There are inconsistencies in the appearance of a number of panels, especially in Fig. 2, which are reported to be from the same experiments. There are also many instances where control panels have been duplicated across different figures and experiments, for example:

The Lamin B1 (HeLa) control panels in Fig. 1, Fig. 2B and C are all identical.

The Lamin B1 (CaSki) control panels in Fig. 2B and C are also identical.

The Lamin B1 panels in Fig. 3 and 4 appear to be identical images.

The authors were asked to provide the raw data for this article, but did not respond. Given the significance of the concerns about the validity of the data, and the lack of raw data, the findings in this paper are not reliable.

The authors have been informed but have not responded to any correspondence regarding the retraction.

Signed: Laura Fisher, Executive Editor, *RSC Advances*.

Date: 15^th^ January 2021.

## Supplementary Material

